# The Impact of Ethnicity and Age on Distribution of Metastases in Patients with Upper Tract Urothelial Carcinoma: Analysis of SEER Data

**DOI:** 10.3390/biomedicines11071943

**Published:** 2023-07-08

**Authors:** Antonio Tufano, Sisto Perdonà, Pietro Viscuso, Marco Frisenda, Vittorio Canale, Antonio Rossi, Paola Del Prete, Francesco Passaro, Alessandro Calarco

**Affiliations:** 1 Department of Maternal-Infant and Urological Sciences, Policlinico Umberto I Hospital, “Sapienza” Rome University, 00161 Rome, Italy; pietro.viscuso@libero.it (P.V.); marco.frisenda57hu@gmail.com (M.F.); canale.vittorio@gmail.com (V.C.); 2 Uro-Gynecological Department, Istituto Nazionale Tumori di Napoli, IRCCS “G. Pascale”, Via M. Semmola, 80131 Naples, Italy; 3 “Cristo Re” Hospital, 00167 Rome, Italy; antonio.rossicz@gmail.com; 4 Scientific Directorate, Istituto Nazionale Tumori di Napoli, IRCCS “G. Pascale”, Via M. Semmola, 80131 Naples, Italy; p.delprete@istitutotumori.na.it; 5 Department of Neurosciences, Reproductive Sciences and Odontostomatology, University of Naples “Federico II”, 80131 Naples, Italy; francescopassaro1996@gmail.com; 6 Clinica Villa Pia, 00151 Rome, Italy; alecalarco@gmail.com

**Keywords:** metastatic upper tract urothelial carcinoma, upper tract urothelial carcinoma, UTUC, Surveillance Epidemiology and End Results, ethnicity, race, age

## Abstract

Upper tract urothelial carcinoma (UTUC) constitutes a rare and aggressive entity accounting for 5% to 10% of all urothelial tumors. The importance of stratification and disparities according to ethnicity and age has never been tested in a sufficiently large sample of patients with metastatic UTUC (mUTUC). We conducted this study to address this void, and we hypothesized that the distribution of metastases may vary according to age and ethnicity. Within the Surveillance, Epidemiology, and End Results (SEER) database (2004–2016), we identified 1115 patients with mUTUC. The chi-square and *t*-test tests were used to examine statistical significance in terms of proportions and mean differences. A total of 925 (83.0%) patients were Caucasians, while 190 (17.0%) were African Americans. Among both ethnicities, lungs were the most common metastatic site (39.1% vs. 48.9%). Brain metastases were infrequent among both ethnicities (1.2 vs. 2.6%; *p* = 0.13). The trends in the lung metastases decreased with age from 42.3% to 36.6% (*p* = 0.010) among Caucasians, whereas they increased among African Americans from 34.0% to 51.7% (*p* = 0.04). Overall, 32.8% of Caucasians and 40.5% of African Americans exhibited more than one metastatic site. Among Caucasians, increasing age was associated with lower rates of having multiple metastatic sites (from 34.3% to 30.2%) (*p* = 0.004). According to our multivariable analyses, younger age was associated with an increased risk of lung (OR: 1.29, 95% CI 1.04–1.71; *p* = 0.045) and bone metastases (OR: 1.34, 95% CI 1.07–1.79; *p* = 0.046). Racial differences exist in the distribution of mUTUC metastasis and vary according to age. Our findings may also be considered in the design of randomized trials.

## 1. Introduction

Upper tract urothelial carcinoma (UTUC) originates in the ureter or renal pelvis and is a seldom encountered yet highly aggressive condition, accounting for 5% to 10% of all urothelial tumors [1,2]. At the time of diagnosis, the distribution of patients with UTUC includes approximately 40–50% of patients with non-muscle invasive UTUC (categorized as pTa/T1), 50–60% of patients with muscle-invasive or non-organ-confined disease (*p* ≥ T2), and up to 25% of patients are diagnosed with metastatic disease [3,4]. Interestingly, the incidence of localized UTUC upon diagnosis is declining, and the presence of distant disease serves as a significant factor affecting both cancer-specific mortality (CSM) and overall mortality (OM) among UTUC patients. 

Metastatic UTUC (mUTUC) typically spreads to regional lymph nodes or distant organs such as the liver, lungs, bones, and other sites. To date, knowledge regarding the distribution of metastases is of paramount importance for the accurate staging and treatment of advanced tumors [5,6,7]. Moreover, previous authors have underlined the importance of stratification according to ethnicity and age with respect to the metastatic distribution and oncological outcomes in several cancers. Specifically, a younger age is often associated with aggressive tumor dissemination [8,9]. This correlation has been recently described in a large metastatic renal cell carcinoma population [10]. At the same, based on epidemiological studies, ethnicity has been shown to affect tumor characteristics and survival in non-metastatic UTUC patients [11]. The reasons for this are motley, and a growing body of research supports the existence of a multifactorial influence on race disparities. Specifically, genetic, societal, and environmental factor differences may partially explain these disproportions [12,13,14]. However, when focusing on mUTUC patients, the effect of ethnicity and age on the distribution of metastases has never been tested in a sufficiently large sample cohort. Therefore, we addressed this void by examining this association in a US-population-based cohort. 

## 2. Materials and Methods

### 2.1. Study Population and Variable Definitions

After scouring the Surveillance, Epidemiology, and End Results (SEER) database (2004–2016), we identified US patients diagnosed with urothelial mUTUC and grouped them according to their ethnicity. We included patients older than 18 years of age meeting the following requirements: a primary diagnosis of mUTUC; pathologically confirmed mUTUC of the renal pelvis, ureter, or ureter orifice (International Classification of Disease for Oncology (ICD-O) site codes C65.9, C66.9, and C67.6, respectively); and confirmed information on metastases in the bones, liver, lungs, brain, or distant lymph nodes. Given the limited representation of other ethnic groups in our sample, which precluded any possibility of obtaining meaningful conclusions, our analyses focused on the differences between Caucasian and African American ethnicities. The analyzed patient characteristics included age, which was coded as a continuous variable and, subsequently, categorized into quartiles: ≤63, 64–72, 73–79, and ≥80 years. Variables such as age, sex, ethnicity, marital status (married vs. unmarried vs. divorced/widowed vs. unknown), region, primary tumor site (renal pelvis vs. ureteral vs. ureter orifice), tumor grade (high grade vs. low grade), T-stage (T1–T2 vs. T3–T4), regional N status (N− vs. N+), and treatment history regarding chemotherapy (not performed/unknown vs. performed) and surgery (not performed/unknown vs. performed) were included. Exclusion criteria were as follows: “other” ethnicities due to the small sample size, patients with unknown age, and patients with unknown metastatic sites.

### 2.2. Statistical Analysis

Means with standard deviations were used to report continuous variables, while frequencies and percentages were used to describe categorical variables. The statistical analysis consisted of 4 parts. First, the chi-square test and *t*-test were used to assess whether differences between proportions in Caucasian vs. African Americans were statistically significant. Second, we explored the rates of site-specific metastasis among the two ethnic groups. Third, we examined the influence of age on the distribution of the number of metastatic sites and on single-organ metastatic sites, analyzing trends in proportions within both ethnic groups. Fourth, multivariable logistic regression models were used to investigate predictors of lung, distant lymph node, bone, and liver metastases. The covariates used in the multivariable logistic regression models were ethnicity (African American vs. Caucasian), age (<65 vs. ≥65 years), and gender (male vs. female). For all statistical analyses, Statistical Package for Social Sciences (SPSS) software v.26.0 (IBM Corp, Armonk, NY, USA) was used. All tests were two sided with a level of significance set at *p* < 0.05.

## 3. Results

### 3.1. Characteristics of the Study Population

Overall, 1115 patients with metastatic UTUC were identified between 2004 and 2016. Of these, 925 (83.0%) were Caucasians, and 190 (17.0%) were African Americans (Table 1). Caucasian patients more frequently presented high tumor grades (77.1 vs. 70.0; *p* = 0.038) and an N+ status (positive regional lymph node status) (69.8% vs. 60.5%; *p* = 0.012) compared to African Americans, whereas no differences were observed for age (*p* = 0.250), gender (*p* = 0.056), primary tumor site (*p* = 0.085), T-stage (*p* = 0.537) surgery (*p* = 0.495), or chemotherapy (*p* = 0.084) between the two ethnicities.

### 3.2. Location of Metastases According to Ethnicity

**Figure 1:**
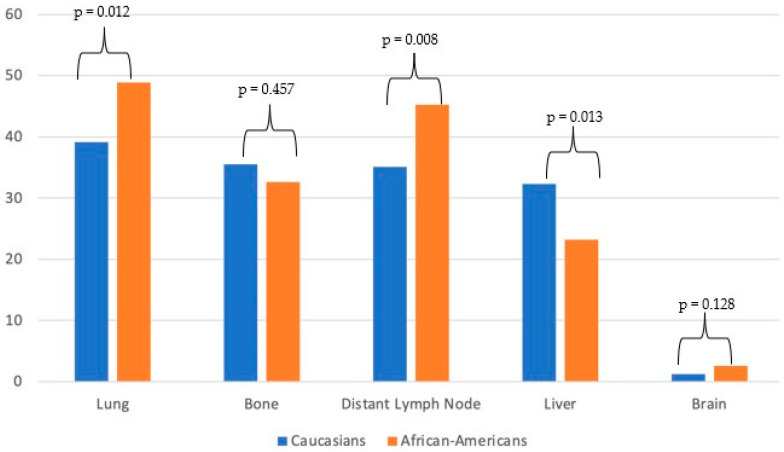
Bar plots depicting rates of metastatic sites in metastatic upper tract urothelial carcinoma patients identified within the Surveillance, Epidemiology, and End Results database between 2004 and 2016 (stratified according to ethnicity).

The rates of site-specific metastasis according to ethnicity are shown in Figure 1. The lungs were observed to be the most common metastatic site among Caucasians (39.1%), followed by bone (35.5%), a distant lymph node (35.1%), and the liver (32.3%). Similarly, among African Americans, the most common metastatic site was a lung (48.9%), followed by a distant lymph node (45.3%), bone (32.6%), and the liver (23.2%). The incidence of brain metastases was found to be low among both Caucasians and African Americans, and no statistically significant difference was observed between the two groups (1.2% vs. 2.6%; *p* = 0.13). Overall, African American patients showed higher rates of lung (*p* = 0.012) and distant lymph node (*p* = 0.008) metastases compared to Caucasians. Conversely, the rates of liver metastases were higher among Caucasian compared to African American patients (*p* = 0.013).

### 3.3. Location of Metastases According to Ethnicity and Age

The rates of site-specific metastases according to ethnicity and age combinations were analyzed (Supplementary Table S1). The rates of lung metastases decreased with age from 42.3% to 36.6% (*p* = 0.010) among Caucasians, while they increased among African Americans from 34.0% to 51.7% (*p* = 0.04). The rates of all other metastatic sites were unaffected by age (all *p* > 0.05). 

### 3.4. Distribution of Single-Organ Metastatic Sites According to Ethnicity and Age

**Figure 2:**
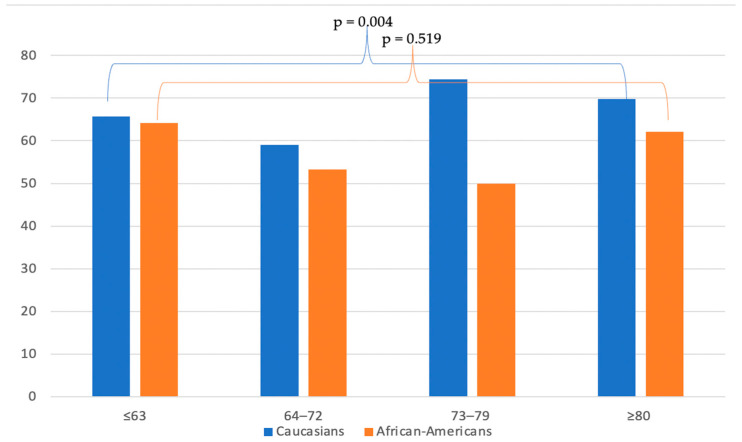
Bar plots depicting rates of single metastasis according to age and ethnicity among upper tract urothelial carcinoma patients identified within the Surveillance, Epidemiology, and End Results database between 2004 and 2016.

Overall, 32.8% Caucasians and 40.5% African Americans exhibited more than one metastatic site (Table 2). Increasing age was associated with lower rates of multiple metastatic sites among Caucasians (from 34.3% to 30.2%) (*p* = 0.004) but not among African Americans (from 35.8% to 37.9%) (*p* = 0.519) (Figure 2). 

**Figure 3:**
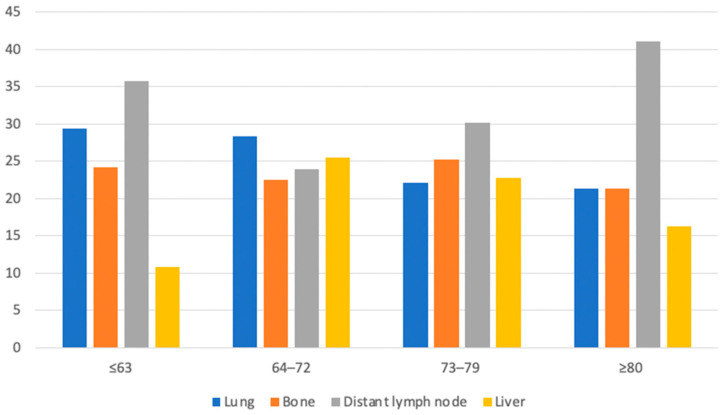
Bar plots depicting rates of single metastasis according to age in Caucasian upper tract urothelial carcinoma patients identified within the Surveillance, Epidemiology, and End Results database between 2004 and 2016.

Caucasians represented the largest sample size in our dataset; therefore, we focused on the detailed distribution of single-organ metastases according to age in this sub-cohort. Important differences were noted. The presence of exclusively distant lymph node or liver metastases increased with age from 35.7% to 41.1% and from 10.8% to 16.3%, respectively (Figure 3). Conversely, the presence of solely lung metastases decreased with age (from 29.3% to 21.3%). Lastly, a more subtle difference was noted among patients with solely bone metastases after age stratification (from 24.2% to 21.3%).

### 3.5. Multivariable Analyses Predicting Lung, Distant Lymph Node, Bone, and Liver Metastases

Lastly, we validated our observations using multivariable analyses (Table 3). According to our multivariable logistic regression models, African American ethnicity presented a higher risk of lung (OR: 1.50, 95% CI 1.08–2.06; *p* = 0.014) and distant lymph node metastases (OR: 1.49, 95% CI 1.08–2.01; *p* = 0.016), but not a higher risk of bone (OR: 0.90, 95% CI 0.64–1.26; *p* = 0.544) or liver metastases (OR: 0.63, 95% CI 0.43–0.92; *p* = 0.015). A younger age was also associated with an increased risk of lung (OR: 1.29, 95% CI 1.04–1.71; *p* = 0.045) and bone metastases (OR: 1.34, 95% CI 1.07–1.79; *p* = 0.046).

## 4. Discussion

We hypothesized that ethnicity plays a role in the distribution of metastatic sites among patients with mUTUC. Additionally, we posited that age may contribute to this association. We examined these two hypotheses within a large, contemporary North American epidemiological database (SEER). To the best of our knowledge, we are not aware of similar studies. Several of our findings are noteworthy. 

First, mUTUC represents a rare entity [15]. The SEER database provides one of the largest sample sizes of mUTUC patients for the purpose of large-scale epidemiological analyses. Due to the insufficient numbers of other ethnic groups in this dataset, thereby precluding any possibility of drawing meaningful conclusions, our analyses solely focused on the differences between Caucasian and African American ethnicities. In our mUTUC SEER population, Caucasians accounted for 83%, and African Americans accounted for 17%. These proportions are consistent with a previous study addressing UTUC incidence according to race/ethnicity [16]. To the best of our knowledge, the reason behind this discrepancy in prevalence is unknown. However, it should be noted that this observation highlights the challenges associated with testing for racial differences in terms of mUTUC. This limitation has also impacted several prior publications on the topic [17,18]. 

Second, among both Caucasians and African Americans, the lung was identified as the most frequent site of metastasis. The significance of chest imaging in urothelial cancer treatment has been firmly established, and international guidelines advocate for the use of chest imaging in staging and follow-ups for patients with bladder cancer and UTUC [19]. 

Bone metastases ranked second highest in prevalence among Caucasian patients (32.6%) and were associated with a younger age (<65 years) according to our multivariable analyses (OR: 1.34; *p* = 0.046). The presence of bone metastases in urothelial cancer is widely recognized as an indicator of unfavorable oncological outcomes. Therefore, it is important to emphasize that early detection can facilitate prompt medical or surgical intervention and help prevent skeletal-related events [20,21]. It is worth mentioning that the NCCN guidelines recommend screening for bone metastasis in specific situations, including in cases of bone-related symptoms, high-risk disease, or when laboratory tests suggest the presence of bone lesions [22]. In light of our findings, routine bone surveys may be useful. Alternatively, bone scans may be used. Taken together, the results of our outcome indicate the need for a reassessment of the selection criteria for bone imaging among UTUC cancer patients. Based on our results, we suggest that a bone scan should be considered for a broader cohort of patients with UTUC.

Third, we hypothesized that age may further affect, in addition to existing racial differences, the distribution of UTUC metastatic sites. Among the observed variations, the most notable disparities were observed in the rates of lung metastases. These rates decreased with increasing age among Caucasians, namely, from 42.3% to 36.6% (*p* = 0.010), whereas they increased among African Americans, rising from 34.0% to 51.7% (*p* = 0.04). Other age-specific racial differences were more subtle in magnitude. Hence, the proportions of these differences might be of importance in the planning and design of clinical trials. However, we did not adjust our analyses for some potential determinant predictive variables, such as socioeconomic status or the possession of insurance, due to database limitations. Additional analyses are warranted to determine whether these differences are attributable to genetic variations or barriers related to access to healthcare.

Fourth, we found that the overall rate of metastases at single vs. multiple concomitant metastatic sites demonstrated an age-related distribution among Caucasian patients. Specifically, younger Caucasian individuals had higher rates of multiple concomitant metastatic sites (34.3% vs. 30.3%; *p* = 0.004). These findings are in accordance with previously published studies on metastatic urothelial cancer [23]. The reason behind this finding could be that younger patients might be correlated with more aggressive genomic alterations and probably because they receive more aggressive treatment than older patients and thus have a longer time until they develop additional metastases. From a practical standpoint, this indicates that younger hospitalized patients with mUCUB are more prone to having multiple sites of metastatic disease, regardless of the specific tabulation scheme utilized. Consequently, it is advisable to conduct more comprehensive imaging studies on these individuals. 

Finally, we investigated the incidence of brain metastases in relation to the presence or absence of multiple concurrent metastatic sites. The overall rate of brain metastases was 1.4%, with no difference between Caucasians and African Americans (*p* = 0.13). These data confirm that central nervous system metastases among patients with urothelial carcinoma are extremely uncommon. These results are in agreement with the findings reported by Rosiello et al., where no meaningful differences based on patient ethnicity were reported among metastatic bladder cancer patients [18]. Nonetheless, these low rates suggest that routine brain imaging may not be required, unless it is based on an elevated level of clinical suspicion [19,20].

The present study exhibits notable strengths, including a robust sample size and the exceptional data quality of the SEER database. Generally, the SEER program collects cases from 18 registries covering approximately 27.8% of the U.S. population. Therefore, the findings from this population-based study are more likely to be representative and generalizable to a larger population than those from single-center studies. However, we also want to acknowledge the following limitations. First, certain variables, known to serve as prognostic factors for lung cancer, were not available within the SEER database, including factors like cigarette smoking and comorbidities. Second, comprehensive details regarding therapeutic information such as treatment with chemotherapy, radiotherapy, and targeted therapies were lacking in the SEER database, which could have influenced or skewed our findings. Third, since all the patients of this study were from the US, the generalizability of our findings to other countries may be limited. Fourth, it is important to note that the SEER database lacks detailed information regarding tumor characteristics and histology. Likewise, the database does not provide data on the specific number of metastases within each metastatic site. Finally, metastases in uncommon metastatic sites were not evaluated in this study.

## 5. Conclusions

Distinct racial disparities are evident in the distribution of mUTUC metastases. The lung was revealed to be the most common metastatic site among both Caucasians and African Americans. However, the distribution and the number of metastases vary according to age. The brain was confirmed to be a rare metastatic site among both ethnicities and thus routine brain imaging may not be warranted. Lastly, our findings may also be incorporated in considerations regarding the design of randomized trials.

## Figures and Tables

**Table 1:** Descriptive characteristics of metastatic upper tract urothelial carcinoma (UTUC) patients.

		Overall 1115	Caucasian 925 (83%)	African- American 190 (17%)	*p* Value
Age, years, mean (SD) ^1^	-	70.5 (10.4)	70.3 (10.4)	71.4 (10.1)	0.250
Sex, n (%)	Male	639 (57.3%)	542 (58.6%)	97 (51.1%)	0.056
Marital Status, n (%)	Married Unmarried Divorced/Widowed Unknown	655 (58.7%) 132 (11.8%) 286 (25.7%) 42 (3.8%)	553 (59.8%) 98 (10.6%) 240 (25.9%) 34 (3.7%)	102 (53.7%) 34 (17.9%) 46 (24.2%) 8 (4.2%)	**0.016**
Region, n (%)	Large Metro County Medium Metro County Non-metropolitan Counties Small Metro County Unknown County	671 (60.2%) 219 (19.7%) 138 (12.4%) 87 (7.8%) (0%)	561 (60.6%) 183 (19.8%) 114 (12.3%) 68 (7.4%) (0%)	113 (59.5%) 38 (20.1%) 23 (12.1%) 16 (8.4%) (0%)	0.162
Primary tumor site, n (%)	Renal Pelvis Ureter Ureter Orifice	763 (68.4%) 295 (26.5%) 57 (5.1%)	622 (67.2%) 257 (27.8%) 46 (5.0%)	141 (74.2%) 38 (20.0%) 11 (5.8%)	0.085
Grade, n (%)	Low High	269 (24.1%) 846 (75.9%)	212 (22.9%) 713 (77.1%)	57 (30.0%) 133 (70.0%)	**0.038**
T-stage, n (%)	<T3 ≥T3	399 (35.8%) 716 (64.2%)	327.4 (35.4%) 598 (64.6%)	118 (61.9%) 72 (38.1%)	0.537
Regional lymph nodes, n (%)	N+	761 (68.3%)	646 (69.8%)	115 (60.5%)	**0.012**
Chemotherapy, n (%)	Performed No/Unknown	580 (52.0%) 535 (48.0%)	492 (53.2%) 433 (46.8%)	88 (46.3%) 102 (53.7%)	0.084
Surgery, n (%)	Performed No/Unknown	429 (38.5%) 686 (61.5%)	365 (39.5%) 560 (60.5%)	70 (37.0%) 120 (63.0%)	0.495

^1^ SD: standard deviation. bold data are statistically significant (*p* < 0.05).

**Table 2:** Distribution of metastases among patients with a single vs. multiple concomitant sites stratified by age and ethnicity.

	Caucasian						African-American					
	Overall	<64	64–72	73–79	≥80	*p* Value	Overall	<64	64–72	73–79	≥80	*p* Value
Single Site, (%)	67.2%	65.7%	59.0%	74.4%	69.8%	**0.004**	59.5%	64.2%	53.3%	50.0%	62.1%	0.519
≥2 Sites, (%)	32.8%	34.3%	41.0%	25.6%	30.2%		40.5%	35.8%	46.7%	50.0%	37.9%	

bold data are statistically significant (*p* < 0.05).

**Table 3:** Multivariable logistic regression predicting lung, distant lymph node, bone and liver metastases in upper tract urothelial carcinoma (UTUC) patients.

	Lung		Distant Lymph Node		Bone		Liver	
	OR (95% CI)	*p* Value	OR (95% CI)	*p* Value	OR (95% CI)	*p* Value	OR (95% CI)	*p* Value
**Ethnicity** Caucasian African-American	Reference 1.50 (1.08–2.06)	**0.014**	Reference 1.49 (1.08–2.15)	**0.016**	Reference 0.90 (0.64–1.26)	0.544	Reference 0.63 (0.43–0.92)	**0.015**
**Age** ≥65 <65	Reference 1.29 (1.04–1.71)	**0.045**	Reference 1.01 (0.75–1.33)	0.982	Reference 1.34 (1.07–1.79)	**0.046**	Reference 0.96 (0.71–1.29)	0.782
**Gender** Female Male	Reference 0.87 (0.68–1.13)	0.267	Reference 1.01 (0.79–1.30)	0.927	Reference 1.31 (1.02–1.70)	0.036	Reference 0.79 (0.61–1.04)	0.90

bold data are statistically significant (*p* < 0.05).

## Data Availability

https://seer.cancer.gov, accessed on 1 July 2022.

## Supplementary Materials

The following supporting information can be downloaded at: https://www.mdpi.com/article/10.3390/biomedicines11071943/s1, Table S1: Location of metastases according to ethnicity and age.
