# Supplementary material for: The Impact of Ethnicity and Age on Distribution of Metastases in Patients with Upper Tract Urothelial Carcinoma: Analysis of SEER Data
Source: Biomedicines. 2023 Jul 8;11(7):1943. doi: 10.3390/biomedicines11071943 (PMC10377577; doi:10.3390/biomedicines11071943)
Supplement: Supplementary file 1 [file biomedicines-11-01943-s001.zip › biomedicines-2459335-supplementary.pdf]

Supplementary Table S1. Location of metastases according to ethnicity and age

	Caucasians					African-Americans				
	≤63	64–72	73–79	≥80	p value	≤63	64–72	73–79	≥80	p value
Lung	42.3%	44.9%	30.6%	36.6%	0.010	34%	60%	53.8%	51.7%	0.04
Distant Lymph Node	33.9%	32.5%	36.5%	40.1%	0.36	54.7%	37.8%	50%	45.1%	0.27
Bone	37.2%	39.7%	33.3%	31.7%	0.27	37.7%	33.3.%	34.6%	27.6%	0.72
Liver	30.5%	37.6%	32.0%	26.7%	0.10	24.5%	22.2%	11.5%	27.6%	0.44
Brain	1.7%	0%	1.8%	1.5%	0.25	0%	2.2%	7.7%	3.4%	0.25
